# Navigating the Misdiagnosis of Pityriasis Rubra Pilaris and Successful Treatment With Guselkumab: A Case Report of Dual Biologic Therapy

**DOI:** 10.7759/cureus.98267

**Published:** 2025-12-01

**Authors:** Danika Bongfeldt, Sylvia Martinez-Cabriales

**Affiliations:** 1 Dermatology, Northern Ontario School of Medicine (NOSM) University, Sudbury, CAN

**Keywords:** dual biologic therapy, dupilumab, guselkumab, pityriasis rubra pilaris, psoriasis

## Abstract

Pityriasis rubra pilaris (PRP) is a rare and chronic dermatologic condition often misdiagnosed due to its clinical resemblance to psoriasis. It significantly impacts quality of life due to its clinical presentation and systemic discomfort. Conventional treatments, including retinoids, corticosteroids, and methotrexate, often yield suboptimal outcomes, particularly in refractory cases. Recently, biologic therapies have shown promise for PRP management. This case report describes the successful treatment of PRP with guselkumab.

A 57-year-old male presented with an eight-year history of refractory papulosquamous disorder affecting 70% of his body surface area, initially diagnosed and treated as psoriasis. The patient had failed topical steroids, topical calcineurin inhibitors, sulfasalazine, and methotrexate. He had a past medical history of asthma treated with dupilumab. On skin exam, there were orange-red scaly plaques on the scalp, trunk, upper and lower extremities, buttocks, and genitals. Some of the plaques coalesced in areas to confluence, making PRP a presumptive diagnosis. Diagnosis was confirmed by three punch biopsies, and treatment with guselkumab 100mg subcutaneous injection was initiated. The patient noted improvement after the first loading dose and achieved complete skin clearance following the second dose four weeks later. At his one-year follow-up, he maintained full clearance while receiving guselkumab 100 mg subcutaneously every eight weeks.

This case highlights the potential of guselkumab as an effective treatment for refractory PRP, even in cases of extensive disease. It underscores the importance of accurate diagnosis through biopsy and demonstrates the feasibility of concurrent biologic therapies for managing comorbid inflammatory conditions. Further research is needed to optimize biologic treatment strategies and enhance understanding of dual biologic use in complex dermatologic cases.

## Introduction

Pityriasis rubra pilaris (PRP) is a rare, chronic skin disorder that is commonly misdiagnosed due to its rarity and resemblance to other skin conditions [1,2]. PRP has a profound impact on an individual’s quality of life, with symptoms including discomfort, poor sleep, itching, anhidrosis, body temperature dysregulation, and self-consciousness [3]. Its therapeutic guidelines are limited: current conventional treatments include retinoids, phototherapy, corticosteroids, and methotrexate [1]. Recently, off-label biologic therapies have been effective in managing PRP; however, this data is limited to mostly case reports and case series [1,4,5]. Therefore, the goal of this case report is to contribute more data regarding the off-label use of biologics in managing PRP.

This article was previously presented as a meeting abstract at the 2025 100th Canadian Dermatology Association Annual Conference on June 18, 2025.

## Case presentation

A 57-year-old male presented for assessment of severe psoriasis diagnosed eight years ago. The onset of his lesions started 20 years ago. He had failed topical steroids, sulfasalazine 1.5 g daily, and four months of methotrexate 25 mg weekly. He had a seronegative spondyloarthropathy (negative Human Leukocyte Antigen-B27 and normal ranges of rheumatoid factor, C-reactive protein, and erythrocyte sedimentation rate) without erosive changes. His comorbidities included asthma, for which he had been on dupilumab 300mg every two weeks for 10 weeks, along with multiple courses of prednisone in the last two years. The withdrawal of prednisone would cause exacerbation of his skin lesions. The patient also experienced anxiety, gastroesophageal reflux disease, hypertension, hyperlipidemia, peripheral vascular disease, erectile dysfunction, and osteopenia. Current active medications included dupilumab, salbutamol, escitalopram, rosuvastatin, perindopril, celecoxib, calcium carbonate, cholecalciferol, and sildenafil. There was no reported family history of any skin conditions.

On skin exam, he had orange-red scaly plaques on the scalp, trunk, upper and lower extremities, buttocks, and genitals, covering a body surface area of 70%. Some of the plaques coalesced into areas of confluence, and there were islands of sparing and follicular hyperkeratotic papules. There was hyperkeratosis of the palms and soles, but fingernails and toenails were clear. The patient’s clinical features of orange-red scaley plaques with distinctive areas of sparing were strongly suggestive of PRP rather than his initial diagnosis of psoriasis. Three punch biopsies were performed to rule out mainly psoriasis, but also other papulosquamous differentials, including atopic dermatitis, sarcoidosis, cutaneous lupus, and cutaneous T cell lymphoma. The punch biopsies showed a thick stratum corneum with alternating areas of hyperkeratosis and parakeratosis, and a granular cell layer. The dermis showed mostly a perivascular lymphohistiocytic infiltrate with mild telangiectasis. Follicular plugging was focally seen. Direct immunofluorescence was negative. Taken altogether, the pathology confirmed PRP.

A pre-immunosuppressive workup was performed with normal results, and the patient’s vaccines were updated as per his age. Treatment with guselkumab 100 mg subcutaneous injection was initiated through the standard loading doses. The patient noted improvement after the first dose and achieved complete skin clearance following the second dose four weeks later. At his one-year follow-up, he maintained full clearance while receiving guselkumab 100 mg subcutaneously every eight weeks.

## Discussion

Guselkumab is a human monoclonal IgG antibody that selectively targets the p19 subunit of interleukin 23 [6]. This case documents remission of PRP with guselkumab within eight weeks and maintained a complete response seven months afterward. Multiple biologics have been used for PRP, including adalimumab, secukinumab, infliximab, ustekinumab, etanercept, ixekizumab, risankizumab, and guselkumab [4-7]. A recent review reported biologics as the most effective therapy for refractory PRP, since almost 70% of PRP cases were determined to be refractory to non-biologic systemic therapies [4]. The greatest resolution has been found with infliximab, followed by ustekinumab, etanercept, and secukinumab [8]. A recent single-arm, non-randomized interventional study found that patients with moderate to severe PRP showed a significant improvement in pruritus and Dermatology Life Quality Index scores after 24 weeks of treatment [6]. Similar to ours and other case report observations, they concluded that guselkumab may be an effective treatment for refractory moderate to severe PRP [6,9,10].

At the time of starting guselkumab, the patient had been on dupilumab for 12 weeks. After seven months, no interactions were presented, and the patient tolerated both biologics. Our case supports dual biologic therapy in managing two separate chronic inflammatory diseases. This case represents one of the first reports of safe off-label dual biologic therapy using guselkumab for PRP and dupilumab for asthma, contributing to the limited literature on concurrent biologic use to manage coexisting inflammatory conditions [11].

This case highlights the common misdiagnosis of PRP due to its resemblance to other papulosquamous skin conditions [1,2,12]. As in our patient, PRP is most commonly misdiagnosed as psoriasis because both present with erythematous, scaly plaques and may involve the scalp, nails, and palms/soles [1,13]. However, PRP typically shows follicular hyperkeratotic papules, orange-red plaques with islands of sparing, and diffuse waxy palmoplantar keratoderma with less adherent scale [14]. In contrast, psoriasis presents with well-demarcated, symmetric plaques with silvery scale, often on extensor surfaces, and classic signs such as the Auspitz sign and Koebner phenomenon [15]. Pathology confirmation is often required to differentiate PRP from psoriasis [13]. On histopathology, PRP presents with variable acanthosis and follicular plugging in the epidermis, vascularity in the dermis, perivascular pattern of inflammation, and lymphocyte infiltration as seen with our patient [1,16].

There are six clinical subtypes of PRP, and our patient had Type II, known as atypical adult type, which has a more chronic course and is found in only 4% of patients [13,14].

## Conclusions

In conclusion, we present the successful management of PRP with guselkumab despite extensive skin involvement. This case highlights the importance of thorough differential diagnoses and punch biopsy to prevent the misdiagnosis of PRP with other papulosquamous disorders like psoriasis. It also reports the safe and successful concomitant use of guselkumab and dupilumab prescribed for PRP and asthma, respectively. This case demonstrates that dual biologic therapy can be successfully employed for managing separate comorbid inflammatory diseases, emphasizing the need for further research and individualized patient care.
